# Proteomic profiling demonstrates inflammatory and endotheliopathy signatures associated with impaired cardiopulmonary exercise hemodynamic profile in Post Acute Sequelae of SARS‐CoV‐2 infection (PASC) syndrome

**DOI:** 10.1002/pul2.12220

**Published:** 2023-04-01

**Authors:** Inderjit Singh, Brooks P. Leitner, Yiwei Wang, Hanming Zhang, Phillip Joseph, Denyse D. Lutchmansingh, Mridu Gulati, Jennifer D. Possick, William Damsky, John Hwa, Paul M. Heerdt, Hyung J. Chun

**Affiliations:** ^1^ Department of Medicine, Section of Pulmonary, Critical Care, and Sleep Medicine Yale School of Medicine New Haven Connecticut USA; ^2^ Yale School of Medicine New Haven Connecticut USA; ^3^ Department of Dermatology Yale School of Medicine New Haven Connecticut USA; ^4^ Department of Comparative Medicine Yale School of Medicine New Haven Connecticut USA; ^5^ Department of Medicine, Yale Cardiovascular Research Center, Section of Cardiovascular Medicine Yale School of Medicine New Haven Connecticut USA; ^6^ Department of Anesthesiology, Division of Applied Hemodynamics Yale School of Medicine New Haven Connecticut USA

**Keywords:** invasive CPET, PASC, proteomics

## Abstract

Approximately 50% of patients who recover from the acute SARS‐CoV‐2 experience Post Acute Sequelae of SARS‐CoV‐2 infection (PASC) syndrome. The pathophysiological hallmark of PASC is characterized by impaired system oxygen extraction (EO_2_) on invasive cardiopulmonary exercise test (iCPET). However, the mechanistic insights into impaired EO_2_ remain unclear. We studied 21 consecutive iCPET in PASC patients with unexplained exertional intolerance. PASC patients were dichotomized into mildly reduced (EO_2_peak‐mild) and severely reduced (EO_2_peak‐severe) EO_2_ groups according to the median peak EO_2_ value. Proteomic profiling was performed on mixed venous blood plasma obtained at peak exercise during iCPET. PASC patients as a group exhibited depressed peak exercise aerobic capacity (peak VO_2_; 85 ± 18 vs. 131 ± 45% predicted; *p* = 0.0002) with normal systemic oxygen delivery, DO_2_ (37 ± 9 vs. 42 ± 15 mL/kg/min; *p* = 0.43) and reduced EO_2_ (0.4 ± 0.1 vs. 0.8 ± 0.1; *p* < 0.0001). PASC patients with EO_2_peak‐mild exhibited greater DO_2_ compared to those with EO_2_peak‐severe [42.9 (34.2–41.2) vs. 32.1 (26.8–38.0) mL/kg/min; *p* = 0.01]. The proteins with increased expression in the EO_2_peak‐severe group were involved in inflammatory and fibrotic processes. In the EO_2_peak‐mild group, proteins associated with oxidative phosphorylation and glycogen metabolism were elevated. In PASC patients with impaired EO2, there exist a spectrum of PASC phenotype related to differential aberrant protein expression and cardio‐pulmonary physiologic response. PASC patients with EO_2_peak‐severe exhibit a maladaptive physiologic and proteomic signature consistent with persistent inflammatory state and endothelial dysfunction, while in the EO_2_peak‐mild group, there is enhanced expression of proteins involved in oxidative phosphorylation‐mediated ATP synthesis along with an enhanced cardiopulmonary physiological response.

## INTRODUCTION

More than 50% of patients who survive the acute phase of coronavirus disease 2019 (COVID‐19) infection may exhibit Post Acute Sequelae of severe acute respiratory syndrome Coronavirus‐2 (SARS‐CoV‐2) infection (PASC) syndrome.^1^ Using invasive cardiopulmonary exercise testing (iCPET), our group previously identified a subgroup of PASC patients with persistent exertional intolerance nearly a year after recovery from mild acute illness that was associated with impaired peak systemic oxygen extraction (EO_2_).^2^ Recent proteomic evidence points to the possible contributions of persistent immune system dysregulation in PASC.^3^ However, there are no well‐established correlations between specific proteomic signatures and the pathophysiological hallmark of impaired peak EO_2_ during iCPET in PASC. Accordingly, we sought to integrate our iCPET infrastructure with proteomic profiling to evaluate for aberrations in protein expression amongst PASC patients with persistent exertional intolerance. Such information may provide insights into disease pathophysiology and potentially serve as a biomarker for persistent disease.

## METHODS

We enrolled 21 consecutive PASC patients referred to the Yale New Haven Hospital Pulmonary Vascular Disease Clinic for iCPET evaluation of unexplained exertional intolerance between March 2020 and February 2021. All patients had no demonstrable cardiopulmonary cause for their exertional intolerance evident by conventional clinical testing (including normal resting echocardiogram, noninvasive CPET, chest computed tomography, and lung function tests). PASC patients were dichotomized into mildly reduced (EO_2_peak‐mild) and severely reduced (EO_2_peak‐severe) EO_2_ groups according to the median peak EO_2_ value. Our method for iCPET have been previously described.^2^ Proteomic profiling (Olink Explore 3072 panel; Olink Bioscience) was performed on mixed venous blood plasma obtained at peak exercise during iCPET. Each collected sample was centrifuged, and the resulting plasma supernatant was aliquoted and frozen at −80°C. All subjects had negative SARS‐CoV‐2 polymerase chain reaction testing within 72 h of their procedure.

### Statistical analysis

Exercise hemodynamic values for PASC patients were compared to reference control data from our recent publication^2^ using independent Student *t* test for parametric and Wilcoxon Rank Sum test for nonparametric data. Comparison of systemic oxygen delivery (DO_2_) between EO_2_peak‐mild and EO_2_peak‐severe groups was performed using the Wilcoxon Rank Sum test. Pearson correlation was performed between peak EO_2_ and plasma proteomics at peak exercise. Differential proteome expression analyses were performed between subjects with EO_2_peak‐mild and EO_2_peak‐severe groupings determined by the median split. Log_2_FC > 1 and *p* < 0.05 were used as an exploratory significance threshold. Differentially expressed proteome subsets were subjected to pathway enrichment and gene ontology analyses.

## RESULTS

**Figure 1:**
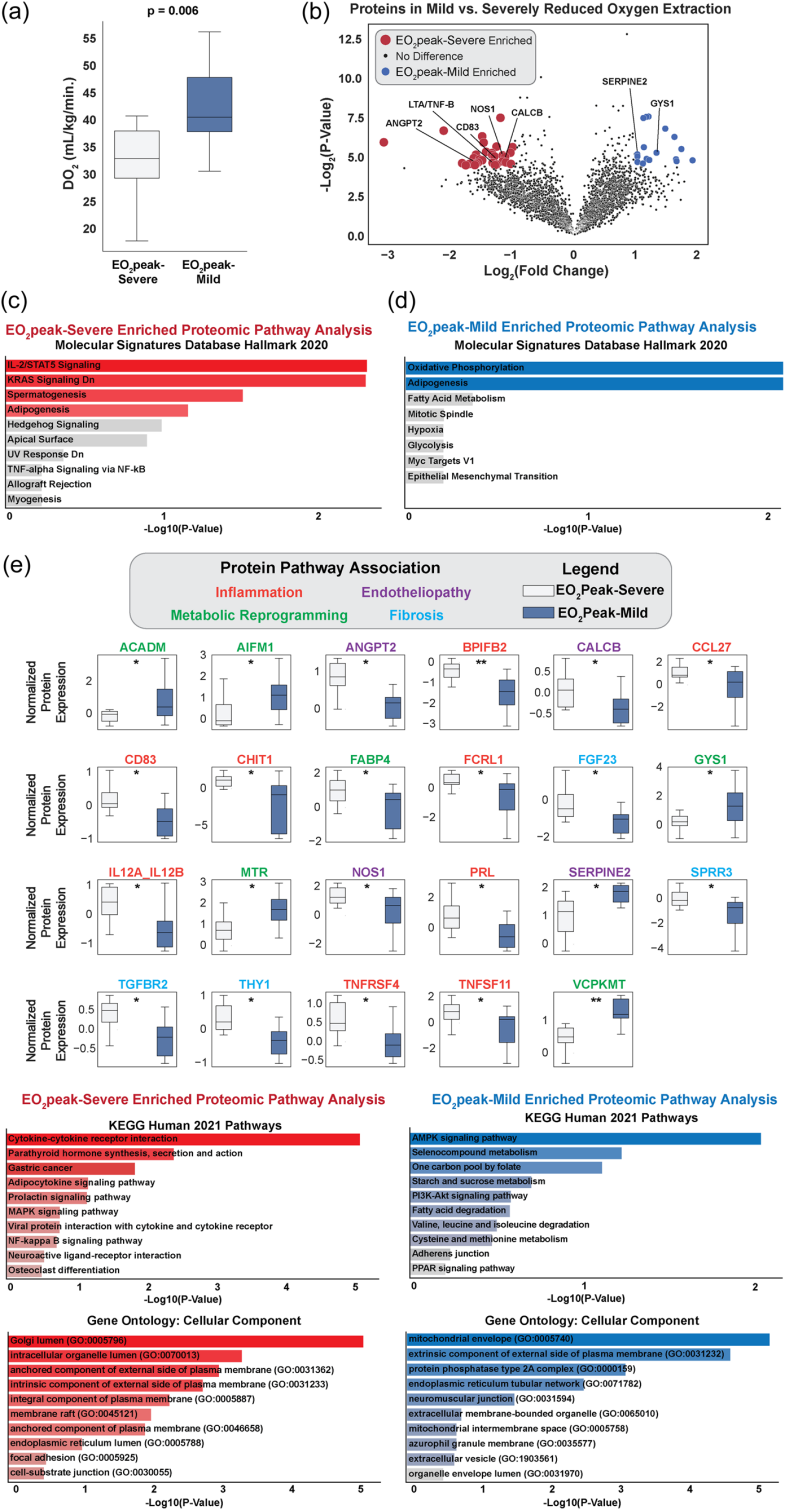
(a) Systemic oxygen delivery (DO_2_) between Post Acute Sequelae of SARS‐CoV‐2 infection (PASC) patients with mild (EO_2_peak‐mild) and severely (EO_2_peak‐severe) reduced peak systemic oxygen extraction (EO_2_) depicted in median and interquartile range. (b) Volcano plot depicting individual proteins expressed in EO_2_peak‐mild and EO_2_peak‐severe PASC groups. (c) Horizontal bar graph depicting enriched proteomic pathway analysis of EO_2_peak‐severe PASC group (blue highlight indicates statistical significance, that is, *p* < 0.05. gray bars represent *p* > 0.05). (d) Horizontal bar graph depicting enriched proteomic pathway analysis of EO_2_peak‐mild PASC group (red highlight indicates statistical significance, that is, *p* < 0.05. Gray bars represent *p* > 0.05). (e) Box plots of individual protein expression between PASC patients with EO_2_peak‐mild and EO_2_peak‐severe impaired peak systemic EO_2_ categorized according to their respective categorical functions of inflammation, metabolic reprogramming, endotheliopathy, or fibrosis (* represents *p* < 0.05 and ** represents *p* < 0.01). Horizontal bar graphs depicting KEGG and Gene Ontology proteomic analysis of EO_2_peak‐mild and EO_2_peak‐severe PASC groups (blue and red highlight indicates statistical significance, that is, *p* < 0.05. Gray bars represent *p* > 0.05). KEGG, Kyoto encyclopedia of genes and genomes.

The baseline characteristics of the enrolled subjects are provided in Table 1. The average age of PASC patients was 50 ± 10 years with 14 out of 21 (66%) being female. PASC patients had an average hemoglobin of 13.5 ± 1.1 g/dL. The interval time from acute COVID‐19 diagnosis to iCPET was 406 ± 130 days. The majority of PASC patients (*n* = 18, 85%) experienced mild acute SARS‐CoV‐2 illness.^4^ PASC patients had normal resting hemodynamics and no exercise PH (peak total pulmonary resistance 1.4 ± 0.6 Woods Unit). PASC patients as a group exhibited depressed peak exercise aerobic capacity (peak VO_2_; 85 ± 18 vs. 131 ± 45% predicted; *p* = 0.0002) with normal DO_2_ (37 ± 9 vs. 42 ± 15 mL/kg/min; *p* = 0.43) and reduced EO_2_ (0.4 ± 0.1 vs. 0.8 ± 0.1; *p* < 0.0001). However, when dichotomized, PASC patients with EO_2_peak‐mild exhibited greater DO_2_ [42.9 (34.2–41.2) vs. 32.1 (26.8–38.0) mL/kg/min; *p* = 0.01] (Figure 1a) and greater cardiac index [9.04 (7.65–11.92) vs. 7.56 (6.62–8.35) L/min/m^2^] compared EO_2_peak‐severe. EO_2_peak‐mild group exhibited reduced EO_2_ compared to controls [0.52 (0.47–0.55) vs. 0.80 (0.76–0.81); *p* < 0.0001]. There was no significant difference in age, female sex distribution, body mass index, and hemoglobin concentration between EO_2_peak‐mild and EO_2_peak‐severe groups. The comparison between EO_2_peak‐mild and EO_2_peak‐severe groups are detailed in Table 1.

**Table 1:** Baseline characteristics and comparison between mild (EO_2_peak‐mild) and severely (EO_2_peak‐severe) reduced peak systemic EO_2_ PASC groups.

Baseline characteristics (*n* = 21)	
Age, years	50 ± 10
Female gender, *n* (%)	14 (66)
Ethnicity (White, Black, Hispanic)	17, 2, 2
BMI (kg/m^2^)	29 ± 7
Hemoglobin (g/dL)	13.5 ± 1.1
Interval time from acute COVID‐19 to iCPET, days	406 ± 130
Severity of acute SARS‐CoV‐2 illness, *n* (%)	
Mild	18 (85)
Moderate	1 (5)
Severe	1 (5)
Critical	1 (5)

Comparison between mild (EO_2_peak‐mild) and severe EO_2_ (EO_2_peak‐severe) groups			
	EO_2_peak‐mild (*n* = 11)	EO_2_peak‐severe (*n* = 10)	*p* Value
Age, years	45 (40–60)	53 (51–59)	0.09
Male gender, *n* (%)	4 (36)	3 (30)	0.75
BMI (kg/m^2^)	27.2 (24.6–29.6)	30.7 (26.6–39.8)	0.10
Hemoglobin (g/dL)	13.7 (13.0–14.6)	12.9 (12.5–14.5)	0.56
Peak Cardiac Index, L/min/m^2^	9.04 (7.65–11.92)	7.56 (6.62– 8.35)	0.03
Peak DO_2_, mL/kg/min	42.9 (34.3–51.2)	32.1 (26.8–38.0)	0.01
Peak CaO_2_, mL/L	186.6 (183.1–207.9)	188.5 (178.7–196.2)	0.77
Systemic oxygen extraction, EO_2_	0.52 (0.47–0.55)	0.40 (0.38–0.42)	<0.0001

*Note*: Data presented as no. (%) or median (interquartile range).

Abbreviations: BMI, body mass index; CaO_2_, oxygen‐carrying capacity in arterial blood; COVID‐19, coronavirus disease 2019; DO_2_, systemic oxygen delivery; EO_2_, oxygen extraction; iCPET, invasive cardiopulmonary exercise test; PASC, Post Acute Sequelae of SARS‐CoV‐2.

Analysis of the proteomic data identified several proteins that were enriched in the EO_2_peak‐mild and EO_2_peak‐severe groups (Figure 1b,e). The top gene‐set enrichment pathway elevated in EO_2_peak‐severe group was the interleukin (IL)‐2/signal transducer and activator of transcription 5 signaling, while the top gene‐set enrichment pathway in the EO_2_peak‐mild group was oxidative phosphorylation (Figure 1c,d). Among the proteins with increased expression in the EO_2_peak‐severe group were proteins involved in inflammatory and fibrotic processes, including tumor necrosis factor (TNF) receptor superfamily 4 (TNFRSF4), transforming growth factor beta receptor 2 (TGFBR2), TNF superfamily‐11 (TNFSF11), TNF‐beta, CC motif chemokine ligand 27 (CCL27), and IL‐12 A/B, as well as angiopoietin‐2 (ANGPT2) is known to be associated with endothelial dysfunction and was previously associated with severe acute COVID‐19.^5^ In the EO_2_peak‐mild group, several proteins associated with oxidative phosphorylation and glycogen metabolism were elevated, including valosin containing protein lysine methyltransferase (VCPKMT), glycogen synthase 1 (GYS1), acyl‐CoA dehydrogenase medium chain (ACADM), and apoptosis inducing factor mitochondria associated‐1 (AIFM1).

## DISCUSSION

In the current study, we demonstrated a spectrum of PASC phenotype with different degree of impaired EO_2_ associated with aberrant protein expression and cardiopulmonary physiologic response. PASC patients with EO_2_peak‐severe appear to exhibit a maladaptive physiologic and proteomic signature consistent with persistent inflammatory state and endothelial dysfunction. To our knowledge, this is the first integration of exercise hemodynamic phenotyping using iCPET and proteomic profiling that provides novel insight into the biological process that may be driving the differentially impaired EO_2_ in PASC.

Consistent with our prior report, we demonstrate that PASC patients with exertional intolerance, but no long‐term cardio‐pulmonary disease sequalae, exhibit reduced peak VO_2_ as a function of impaired EO_2_ relative to controls.^2^ Additionally, we identified several proteins that are differentially expressed according to the severity of impaired EO_2_ including TNF‐beta, which has been previously described in PASC^6^ (Figure 1e). PASC patients with EO_2_peak‐severe demonstrated elevated protein markers that are associated with persistent inflammation and endotheliopathy. Among these include ANGPT2 and IL‐12, both of which are multifaceted factors with pro‐inflammatory and microvascular regulatory properties. Elevated ANGPT2 has been associated with micro‐vascular regression^7^ and systemic capillary rarefaction has been described in PASC.^8^ Elevated IL‐12 has been described in acute SARS‐CoV‐2 infection^9^ and has antiangiogenic properties.^10^ It is therefore plausible that elevated ANGPT2 and IL‐12 associated micro‐circulatory rarefaction in EO_2_peak‐severe group resulted in impaired EO_2_ from a mismatch between microcirculatory perfusion and mitochondrial oxidative metabolism. Additionally, mitochondrial DNA dysregulation^11^ and alteration in systemic vascular compliance^12^ have been observed in autoimmune inflammatory diseases, which may also contribute to impaired EO_2_. The persistent inflammatory response in the EO_2_peak‐severe group resulting in worsen EO_2_ warrants further investigation. Along with this aberrant proteomic profile, we also observed an impaired cardiopulmonary physiological response characterized by reduced DO_2_ in EO_2_peak‐severe group during iCPET (Figure 1a and Table 1). In the EO_2_peak‐mild group, we observed enhanced expression of proteins involved in oxidative phosphorylation‐mediated ATP synthesis (Figure 1d). Additionally, the EO_2_peak‐mild group exhibited an enhanced cardiopulmonary physiological response with greater DO_2_ in response to the reduced peak EO_2_ (Figure 1a). Ultimately, this proteomic reprogramming and augmented physiologic response were insufficient, as overall, the peak EO_2_ in EO_2_peak‐mild group was reduced compared to controls.

Results from the current study need to be interpreted in the context of several limitations. First, data for this study were drawn from a small number of patients who had recovered from acute COVID‐19 illness. However, the impaired EO_2_ and resultant peak VO_2_ exhibited by PASC patients are in keeping with prior reports.^2^, ^13^ Additionally, by combining our iCPET infrastructure with proteomic profiling, we provided a comprehensive and unparalleled insight into the pathophysiological hallmark of exertional intolerance in PASC, that is otherwise not apparent on conventional investigative testing. Second, the current study is devoid of a healthy control group which would ideally consist of individuals with known prior SARS‐CoV‐2 exposure but without exertional intolerance. The prospective accrual of healthy subjects without reported history or documented SARS‐CoV‐2 infection is undoubtedly challenging in the COVID‐19 era. Recruitment of a control cohort was therefore limited, as in our institution, the iCPET represents a clinically indicated study performed in symptomatic patients only.

In conclusion, we demonstrate that in PASC, there is a spectrum of severity related to EO_2_ where patients with EO_2_peak‐severe exhibit a maladaptive cardiopulmonary response as well as persistent inflammatory and endotheliopathy phenotype with resultant impaired EO_2_ and reduced peak VO_2_. Further studies exploring the mechanistic role of these aberrant protein expressions in PASC may help reveal novel pathways which can be leveraged for potential therapeutic opportunities.

## AUTHOR CONTRIBUTIONS

**Inderjit Singh**: Study design, data collection, data analysis, manuscript preparation. **Brooks P. Leitner**: Data analysis and manuscript preparation. **Yiwei Wang**: Data analysis. **Hanming Zhang**: Data collection. **Phillip Joseph**: Data collection and manuscript preparation. **Denyse D. Lutchmansingh**: Data collection. **Mridu Gulati**: Data collection. **Jennifer D. Possick**: Data collection. **William Damsky**: Data analysis. **John Hwa**: Manuscript preparation. **Paul M. Heerdt**: Manuscript preparation. **Hyung J. Chun**: Study design, data analysis, manuscript preparation.

## CONFLICT OF INTEREST STATEMENT

The authors declare no conflict of interest.

## ETHICS STATEMENT

Yale IRB (IRB 2000024570 and IRB 2000024783).
